# Efficacy of floating needles in the treatment of postpartum urinary incontinence and pain: A systematic review and meta-analysis

**DOI:** 10.1097/MD.0000000000048440

**Published:** 2026-04-17

**Authors:** Dongmiao Han, Min Tang, Shuang Gong, Wanyi Li, Zhitao Liu, Zicai Liu

**Affiliations:** a Department of Rehabilitation Therapy Teaching and Research, Gannan Healthcare Vocational College, Jiangxi, Ganzhou, China; b Department of Neurological Rehabilitation II, Ningbo Rehabilitation Hospital, Ningbo, Zhejiang, China; c Department of Rehabilitation Medicine, Shaoguan First People’s Hospital, Guangdong, Shaoguan, China.

**Keywords:** float needle, float therapy, labor pain, meta-analysis, postpartum pain, urinary incontinence

## Abstract

**Background:**

Postpartum pain and urinary incontinence (UI) are the most common postpartum sequelae, which can lead to postpartum anxiety and depression when severe, and seriously affect the quality of life of postpartum mothers. Floating needle (FN) is one of the Chinese medical treatments of the latest in recent years, and studies have shown that FN for postpartum pain and stress urinary incontinence has a positive effect; this study aimed to comprehensively and critically evaluate the available evidence on the effectiveness of FN in managing postpartum pain and UI, and provide the first evidence-based medical evidence for FN intervention for postpartum sequela.

**Methods:**

Computerized retrieval of Chinese databases (CNKI, Wanfang, VIP, and CBM) and English databases (PubMed, Embase, Web of Science, and Cochrane Library) for randomized controlled trials on FN therapy for postpartum pain and UI was conducted up to December 20, 2023. Search terms included “floating needle,” “urinary incontinence,” and “labor pain.” Data extraction and quality assessment were performed on eligible studies. Meta-analysis was conducted using RevMan 5.4, with mean differences (MDs) for continuous outcomes and risk ratios for dichotomous endpoints.

**Results:**

A total of 1194 women were included in 8 articles in both Chinese and English, all of which were conducted in China; 6 of the articles were included in the meta-analysis. The FN is more effective in treating postpartum incontinence and pain than the control group (risk ratio = 1.33, 95% confidence interval [CI] = 1.18–1.5, *P* < .001), and significantly reduced the International Consultation on Incontinence Questionnaire-Short Form score (MD = 1.56, 95% CI = 0.78–2.35, *P* < .001); FN therapy can significantly reduce the amount of urine leakage in postpartum patients (MD = 1.79, 95% CI = 1.09–2.49, *P* < .001).

**Conclusion:**

Our results suggest that FN therapy could be encouraged as one of the routine treatments for postpartum sequelae.

## 1. Introduction

Postpartum pain and urinary incontinence (UI) are health problems associated with poor quality of life for women worldwide and are the most common complications of childbirth. Postpartum pain and UI can negatively impact a woman’s physical, mental, and social health and can impose substantial lifestyle limitations. Stress urinary incontinence (SUI), in which the chief complaint is UI due to effort or physical exertion or sneezing or coughing, is the most common type of UI in pregnant women.^[1]^ The study by Morkved and Bø^[2]^ found that the prevalence of SUI in Norway was 42% during pregnancy and 38% at 8 weeks postpartum. In Jordan,^[3]^ the prevalence of SUI is as high as 45%. In India,^[4]^ the prevalence of SUI during pregnancy was 19.2%. The highest prevalence of SUI was found in the United States. Thomason et al^[5]^ reported as high as 60%, whereas Raza-Khan et al^[6]^ reported a prevalence of UI of 70% and 75% in non-parturient and parturient women, respectively. Such a high prevalence of UI places a serious burden on patients, so it is important to improve the situation of maternal UI and reduce the impact of UI. The etiology of female SUI is multifactorial, and childbirth trauma is one of the most important risk factors for SUI.^[7]^ Many patients with SUI cannot recover after delivery and eventually develop persistent UI.^[8,9]^ Current treatment methods for SUI include surgical treatment and various nonsurgical treatments such as behavioral therapy, pelvic floor muscle exercise (PFME), medication, and other treatments.^[10,11]^ Drug therapy focuses on the bladder and urethral sphincter function. Western medicine therapy for female SUI has significant evidence-based medical evidence and significant effects, but there are also some limitations, such as duloxetine causes central system damage and hepatotoxicity side effects.^[12]^ Long-term estrogen therapy may increase the risk of endometrial, breast, and ovarian cancer.

Currently, many women choose to engage in pelvic floor muscle training or Kegel exercises. PFME,^[1]^ repeated selective autonomic contraction and relaxation of specific pelvic floor muscles, also known as Kegel exercise, is the most popular treatment for SUI. For pregnant women with SUI, PFME is the most commonly used conservative treatment and can reduce the prevalence of UI in the short term.^[13]^ However, women without symptoms of SUI may be reluctant to invest their time in PFME.^[14]^ Therefore, it is difficult to master these movements correctly. We need to find more reliable and faster interventions to treat SUI.

Labor pain and postpartum pain not only affect maternal physical and mental health but also can stimulate a series of reactions in the body. At present, there are many methods to treat pain, which can be divided into drug therapy and nondrug therapy.^[15]^ Nondrug treatments such as relaxation techniques, acupuncture, acupressure therapy, and aromatherapy are available.^[16]^ Floating needle (FN) as a nondrug therapy has been used for labor pain intervention; compared with duloxetine, FN therapy has more rapid analgesia and significantly reduced adverse reactions.^[17]^

FN is through the subcutaneous sweep a wide range of bulk subcutaneous loose connective tissue combined with movement resistance of muscle blood perfusion, again to new and old students a lot of blood and nutrients to transport, metabolic waste out, for the recovery of tissue cells to create a good material foundation and internal environment, help to relieve pain postpartum.^[18]^ FN has a very unique effect in the treatment of various acute and chronic congestion pain and has been widely used in clinical practice.^[19]^ Previous studies on the treatment of SUI by floating acupuncture combined with acupoint burying acupuncture showed that floating acupuncture can effectively intervene in SUI and relieve symptoms of SUI patients.^[20,21]^ Research by Chen et al^[22]^ showed that FN therapy combined with acupoint embedding needle treatment can quickly release the affected muscle, relieve pelvic floor muscle, and enhance muscle tension, thus restoring the urethral support system function and improving urinary leakage.

Although FN therapy has been widely used in postpartum pain and SUI, there is no comprehensive and systematic review to evaluate the effectiveness of FN therapy in alleviating postpartum pain and improving stress UI. This study aimed to comprehensively and critically evaluate the available evidence on the effectiveness of FN in managing postpartum or labor pain and SUI, and provide the first evidence-based medical evidence for FN intervention for postpartum sequela.

## 2. Materials and methods

Systematic evaluation and meta-analysis were conducted according to the Preferred Reporting Items for Systematic reviews and Meta-Analyses. No review protocol was registered in PROSPERO, but we strictly adhered to Preferred Reporting Items for Systematic reviews and Meta-Analyses guidelines to ensure transparency.

### 2.1. Inclusion and exclusion criteria

#### 2.1.1. Type of study

Only randomized controlled trials (RCTs) related to FN intervention for postpartum pain and UI were included, excluding reviews, case reports, quasi-randomized controlled trials, animal studies, or non-randomized trials.

#### 2.1.2. Type of participant

Women with pain during delivery, UI, or pain after delivery were included in this meta-analysis, without first-term or parturient, or country restrictions.

#### 2.1.3. Type of interventions

Studies included FN interventions alone or in combination with any other intervention. Except for the FN intervention in the experimental trial, the interventions in the experimental and comparison trials are the same.

#### 2.1.4. Type of outcome measures

The main outcomes included a 1-hour pad test, visual analogue scale, or numeric rating scale, International Urinary Incontinence Advisory Board Urinary Incontinence Questionnaire (International Consultation on Incontinence Questionnaire-Short Form [IC-IQ-SF]) score, pelvic floor muscle strength, and so on.

### 2.2. Search strategy

All relevant Chinese and English literature was searched by 2 authors (DMH and ZCL). We searched 8 Chinese and English databases. English databases were PubMed, Web of Science, Embase, and the Cochrane Library, and Chinese databases include CNKI, China VIP, CBM, and Wanfang. All searches were conducted in December 2023 and covered the databases from their inception. Search terms include the following: (“urinary incontinence or stress urinary incontinence” OR “pain or Postpartum pain or labor pain”) And (“float needle or float therapy or FN or floating needle”) And (“Postpartum OR woman OR female”).

Using the PubMed database as an example, the search strategy is presented in Table 1.

**Table 1:** The specific search strategy of the PubMed database.

No	Search items
1	“urinary incontinence” [Title/Abstract]
2	“stress urinary incontinence” [Title/Abstract]
3	“SUI” [Title/Abstract]
4	1 or 2 or 3
5	“Postpartum pain” [Title/Abstract]
6	“labor pain” [Title/Abstract]
7	5 or 6
8	4 or 7
9	“float needle or float therapy” [Title/Abstract]
10	“FN” [Title/Abstract]
11	“floating needle” [Title/Abstract]
12	9 or 10 or 11
13	8 and 12

### 2.3. Study selection

The authors looked at the titles, abstracts, and keywords of articles found in an electronic search and excluded irrelevant articles. Then, the full text was obtained; through the full text of the study, the unqualified study was eliminated. All differences and comments were resolved through discussions between the 2 researchers, and the included studies were identified according to the criteria for inclusion. Differences in data extraction were resolved through third parties.

### 2.4. Data extraction

The basic information in Table 1 was extracted from the original studies included, including the first author and year of publication, country, patient sample, age, outcome measures, intervention and control group measures, and follow-up period. Data were extracted independently by 2 authors (DMH and ZCL), and differences between them were resolved by a third party (HYL).

### 2.5. Risk of bias assessment

The risk of bias in the study was assessed separately by the authors (DMH and ZCL) according to the Intervention Systems Evaluation Manual. If there is any disagreement, it can be resolved through discussion or a third party (YH). Review Manager 5.4 software (developed and published by The Cochrane Collaboration, Rigshospitalet, Copenhagen, Denmark) was used to perform a risk bias analysis based on the included studies from 7 risk sources, including random sequence generation, allocation concealment, blinding of participants and personnel, blinding of outcome assessment, incomplete outcome data, selective reporting, and other bias, categorizing risks into high risk, low risk, and uncertain bias risk.

### 2.6. Statistical analysis

Meta-analysis was performed using RevMan 5.4 software. The chi-square test was used to test the heterogeneity of the included studies. The test level is α = 0.05. The degree of heterogeneity was observed according to the *I*^2^ value. If all studies are considered to be homogeneous, a fixed effect model is adopted for analysis. If there is moderate or greater heterogeneity in the study, a random-effects model will be used. In the meta-analysis, mean differences (MDs) were computed for continuous outcomes, and risk ratios for dichotomous endpoints. If necessary, sensitivity analysis will be performed to explore the source of heterogeneity.

## 3. Results

### 3.1. Search results

**Figure 1.**
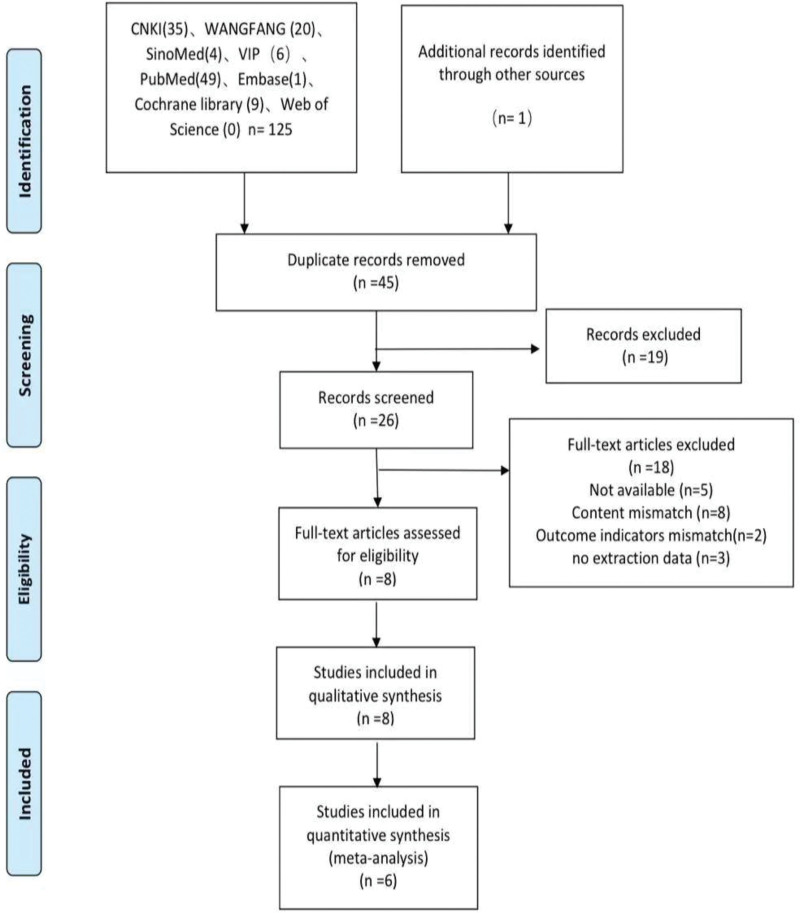
Flowchart of the study search and selection process.

Figure 1 shows the results of the literature retrieved from 8 databases for this review. A database search revealed a total of 89 studies related to FN intervention for postpartum pain or UI. There are 44 abstracts after removing duplicate literature. Reading titles and abstracts excludes 19, leaving 26 to be read in full. Twenty-six studies were excluded after reading the full text, mainly because of inadequate study design, inadequate interventions, or incomplete data. Then, 8 articles were included. Li^[23]^ and Zhu et al^[24]^ only conducted descriptive qualitative analysis but did not conduct a quantitative meta-analysis. Finally, a quantitative meta-analysis was conducted on 6 studies.^[22,25–29]^

### 3.2. Included research characteristics

The 8 included studies were all from China. The baseline characteristics of these studies are shown in Table 2. The study included patients with SUI, postpartum breast congestion, and postpartum pain. The studies included in this meta-analysis included a total of 1194 female patients, 597 assigned to the FN group and 597 to the control group. Their average age is over 29 years.

**Table 2:** Basic information included in the study.

Study	Age	Sample	Patient	Intervention	Follow-up	Outcome
Wang ZX 2021	G1: 50.86 ± 5.68; G2: 49.96 ± 5.11	60	Postpartum SUI	G1: FNT, 100 t/min, sweep for 2 min at each entry G2: PFBT, 30 min/5 times weekly	4 wk	ER/CSS/SF-36/urine leakage/myopotential
Wang ZX 2022	G1: 30.26 ± 6.10; G2: 30.21 ± 6.12	60	Postpartum SUI	G1: FNT, 100 t/min, sweep for 2 min at each entry G2: PFBT, 30 min/5 times weekly	8 wk	ER/urination status/myopotential
Wang XY 2021	G1: 54.39 ± 11.30; G2: 53.41 ± 12.28	60	SUI	G1: FNT, 15–20 min/3 times weekly G2: electroacupuncture, 20 min/5 times weekly	3 wk	ER/1-h pad test/IC-IQ-SF/I-QOL
Fengna Chen 2021	G1: 60.03 ± 2.24; G2: 60.25 ± 2.11	64	Postpartum SUI	G1: FNT, 100 twists per min/2 min G2: drug + Kegel, 2 times/d, 15 min, 8 wk	6 mo	ER/IC-IQ-SF/1-h urine pad test/pelvic floor muscle strength
Chen HJ 2021	G2: 41.50 ± 12.78; G2: 44.40 ± 14.84	60	Postpartum SUI	G1: FNT, 100 t/min, sweep for 2 min at each entry/6 times weekly G2: Kegel motion, 1 time/d, 6 time/wk, 4 wk	4 wk	ER/1-h urine pad test/IC-IQ-SF/pelvic floor muscle strength
Zhu JM 2021	G1:29.63 ± 2.87; G2:28.37 ± 2.34	230	Intrapartum	G1: FNT G2: routine care treatment	After childbirth 6 h	HAMA/blood flow/NAS/EPDS/neonatal Apgar score
Zhang LL 2020	G1: 31.57 ± 4.67; G2: 32.00 ± 4.98	60	Postpartum breast hyperemia	G1: FNT, 100 t/min,0.5 h/d, 3 d G2: acupoint massage, 30–40 min/t, 1 t/d, 3 d	1 mo	ER/breast hardness score/NRS
Li XJ 2013	G1: 41.50 ± 12.78; G2: 27.12 ± 5.19	600	Intrapartum pain	G1: FNT, 3 min G2: nitrous oxide, 4–7 L/min	N	Evaluation criteria of analgesic effect during labor, amount of bleeding during

CSS = clinical symptom score, EPDS = Edinburgh Postnatal Depression Scale, ER = effective ratio, FNT = floating needle therapy, G1 = experimental group, G2 = control, HAMA = Hamilton Anxiety Scale, IC-IQ-SF = International Consultation on Incontinence Questionnaire-Short Form, I-OQL = incontinence quality of life, NAS = neonatal Apgar score, NRS = numeric rating scale, PFBT = pelvic floor biofeedback therapy, SUI = stress urinary incontinence, t = times.

For intervention measures, the operation mode and frequency of the FN were described in detail in all 8 articles. According to the baseline table, the minimum duration of FN intervention was only once at 6 hours postpartum, the longest was 6 months, and most were 3 to 4 weeks. The frequency of FN is 100 times/min, and the sweep time of each entry point is about 2 minutes, usually once every other day (Table 2).

### 3.3. Risk of bias

**Figure 2.**
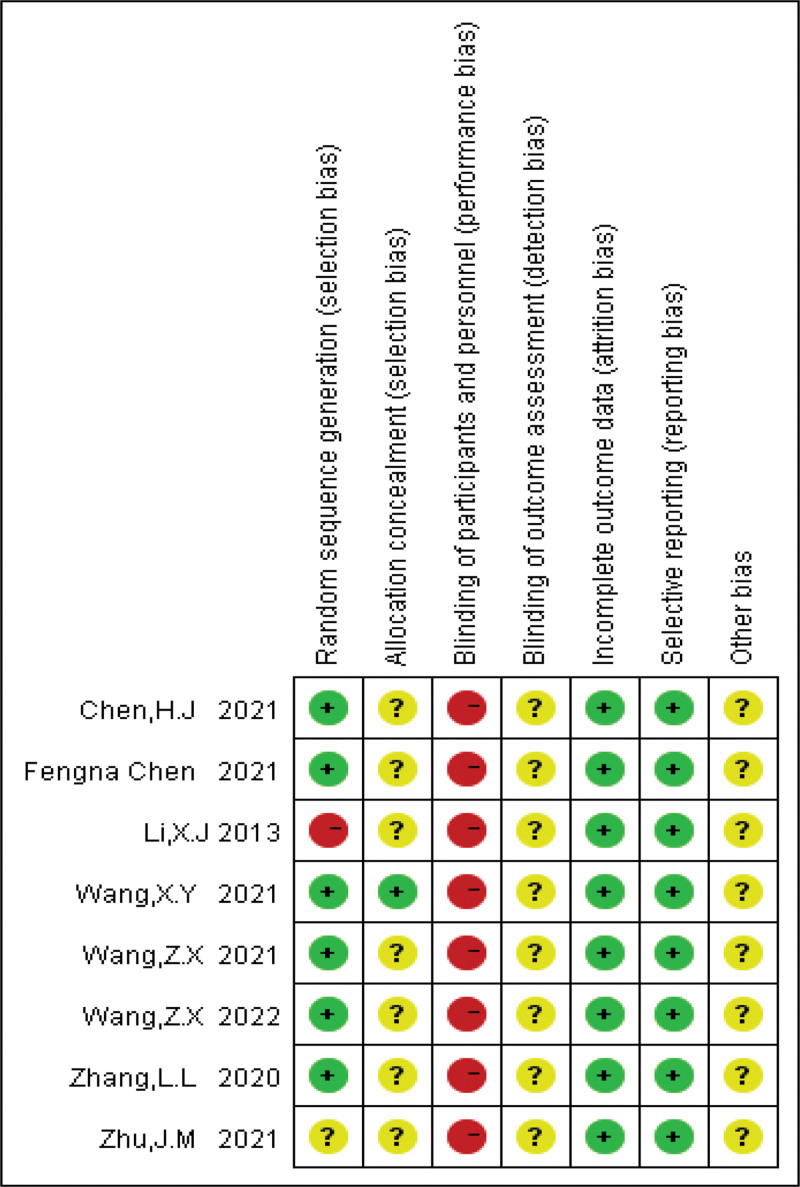
Risk of bias summary: review authors’ judgments about each risk of bias item for each included study.

**Figure 3.**
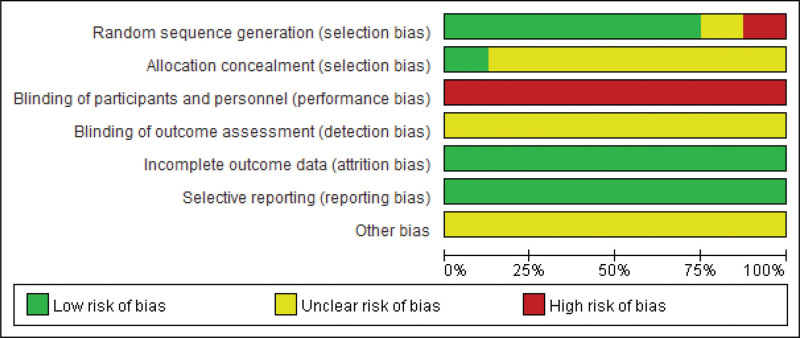
Risk of bias graph: review authors’ judgments about each risk of bias item presented as percentages across all included studies.

We assessed the risk of bias in included studies according to the Cochrane Handbook for Systematic Evaluation of Interventions. The results of the risk of bias are shown in Figures 2 and 3. Six of the 8 studies described the use of a randomized control number table as a method of randomization. The study by Zhu et al^[24]^ did not describe the specific randomization method, so the evaluation was unclear. The study by Li^[23]^ was grouped according to different intervention methods, so it was evaluated as high risk. One detailed the methods for assigning concealment,^[28]^ and 7 studies did not mention detailed blinding and were rated unclear. In the same way that acupuncture does not blind patients, treated patients are aware that they have been treated with FNs, so all studies are at high risk in areas of performance bias. Protocols were available for all studies, and the presented outcomes were reported. The data from these studies are complete without conclusive data. All studies were unclear with respect to other bias and selection bias, and were rated as low risk with respect to reporting bias and attrition bias.

### 3.4. Meta-analysis results

**Figure 4.**
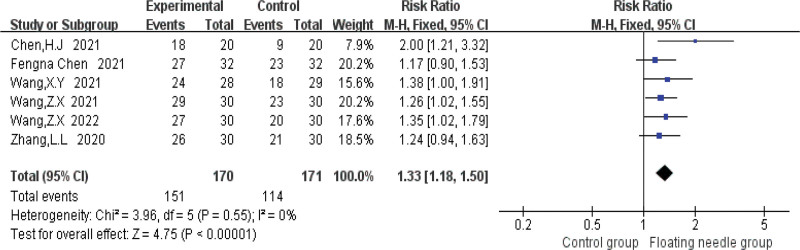
Comparison of the overall efficacy of treating postpartum urinary incontinence and pain forest plot. CI = confidence interval.

**Figure 5.**
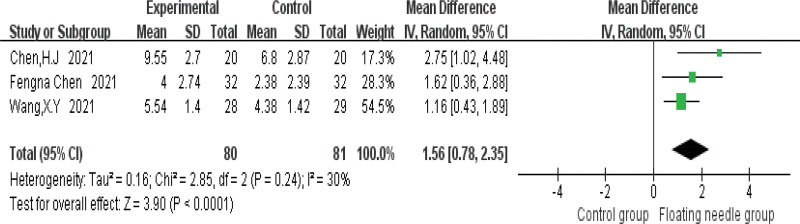
Reduced IC-IQ-SF contrast forest plot. CI = confidence interval, IC-IQ-SF = International Consultation on Incontinence Questionnaire-Short Form, IV = inverse variance, SD = standard deviation.

**Figure 6.**
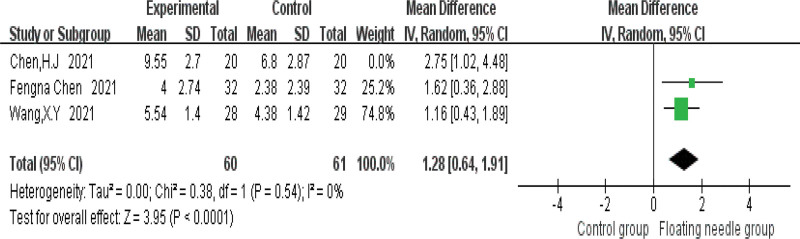
Chen et al study was removed to reduce the comparative forest plot of IC-IQ-SF. CI = confidence interval, IC-IQ-SF = International Consultation on Incontinence Questionnaire-Short Form, IV = inverse variance, SD = standard deviation.

**Figure 7.**
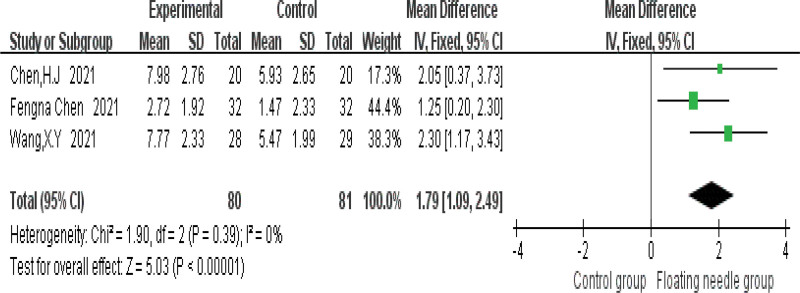
Comparative forest plot for reducing urine leakage. CI = confidence interval, IV = inverse variance, SD = standard deviation.

**Figure 8.**
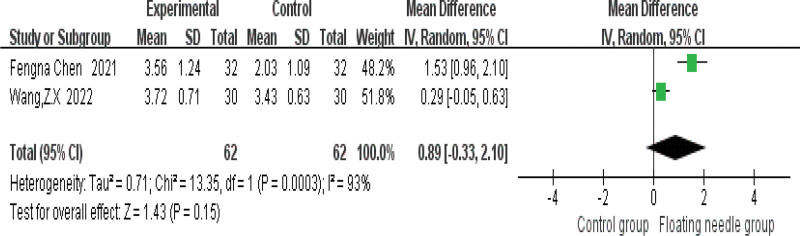
Contrast forest plot of improved pelvic floor muscle strength. CI = confidence interval, IV = inverse variance, SD = standard deviation.

Figure 4 shows that FN is more effective in treating postpartum incontinence and pain than the control group (risk ratio = 1.33, 95% confidence interval [CI] = 1.18–1.5, *P* < .001), and the heterogeneity test (*I*^2^ = 0%, *P* = .55), which means there is no heterogeneity; 3 studies used IC-IQ-SF to evaluate the improvement of FN therapy in postpartum patients. Figure 5 shows that FN therapy can significantly reduce the IC-IQ-SF score of postpartum incontinence and pain patients (MD = 1.56, 95% CI = 0.78–2.35, *P* < .001), with moderate heterogeneity (*I*^2^ = 30%, *P* = .24); the possible sources of heterogeneity were explored through sensitivity analysis, and it was found that the study of Chen et al might be the source of heterogeneity due to its combined intervention of FN with Kegel training, which differed from other studies using FN monotherapy. When this study was deleted, the results remained stable (MD = 1.28, 95% CI = 0.64–1.91, *P* < .001) and no heterogeneity (*I*^2^ = 0%, *P* = .54); compared with the control group, FN significantly reduced IC-IQ-SF score (Fig. 6). Figure 7 shows a comparative forest plot of the 1-hour pad test from 3 studies, and the results showed that FN therapy can significantly reduce the amount of urine leakage in postpartum patients (MD = 1.79, 95% CI = 1.09–2.49, *P* < .001), and no heterogeneity (*I*^2^ = 0%, *P* = .39); the results in Figure 8 show that the FN had no significant difference in improving pelvic floor muscle strength compared with the control group (MD = 0.89, 95% CI = −0.33 to 2.10, *P* = .15), with high heterogeneity (*I*^2^ = 93%, *P* = .0003); small sample size and high heterogeneity limited this conclusion.

### 3.5. GRADE assessment of evidence

The quality of evidence was evaluated using the GRADE framework. Overall evidence was rated as “low” due to high risk of performance bias (lack of blinding in all trials), imprecision (small sample sizes and wide CIs), and indirectness (all studies conducted in China, limiting generalizability). Future high-quality RCTs are needed to strengthen the evidence.

## 4. Discussion

This study is the first systematic meta-analysis of FN intervention for postpartum pain and UI, which has pioneering significance, providing evidence-based medical evidence for FN intervention for postpartum pain and UI, and laying a foundation for related research. Meta-analysis showed that FN therapy had significantly better efficacy in the treatment of postpartum incontinence and pain than the control group, as shown in Figure 4. Figure 5 shows that FN therapy significantly reduced the IC-IQ-SF score of patients with postpartum incontinence and pain, and Figure 7 shows a comparative forest plot of 1-hour pad trials from 3 studies. The results showed that FN therapy could significantly reduce urine leakage in postpartum patients. As shown in Figure 8, FN showed no significant difference in improving pelvic floor muscle strength compared with the control group. Although FNs were not effective in improving pelvic floor muscle strength, they did improve postpartum incontinence and pain to some extent in women, and this may be related to the sample size and heterogeneity of the study.

FN therapy is an invasive physical therapy pioneered by Dr Fu Zhonghua in the clinical practice of acupuncture and moxibustion, mainly using disposable FN tools (publication No. CN1186653A, application No. CN97114318.8), which is a therapeutic tool for the treatment of local symptoms by inserting needles around the pain, with the tip of the needle directed at the lesion and the needle body traveling along with the superficial fascia layer. FN therapy^[30]^ works under the skin, not deep into the muscle layer, like floating on the muscle, because its acupuncture is different from the traditional acupuncture method, so it is named “FN.” Disposable FN is mainly used as the treatment tool to inject needles around the pain. The tip of the needle is aligned with the focus, the needle body moves along the superficial fascia layer, and the left and right sweeping action is carried out. FN therapy^[31]^ is developed based on acupuncture theory, Ahishi acupoint theory, and wrist and ankle acupuncture theory in the Book of Internal Medicine. It mainly treats soft tissue pain diseases and is mostly used for pain syndrome in the clinic. FN therapy is developed based on traditional acupuncture and moxibustion therapy, and its theoretical origin is closely related to traditional acupuncture and moxibustion theory, mainly including the following aspects of the theory^[32,33]^: skin theory, proximal treatment theory, the theory of taking pain as losing, and the stabbing method of “Neijing.” FN treatment of postpartum sequela mechanisms includes^[34,35]^ the following: loose connective tissue theory: the FN extrusion, pulling, and especially sweeping action cause the release of bioelectricity; when the bioelectricity reaches the diseased tissue, the inverse piezoelectric effect is produced, and the internal resistance mechanism of the human body is mobilized, to quickly alleviate the pain; neural theory^[36]^: by directly stimulating the conduction of the pain nerve, the conduction of pain fiber in this kind of nerve is blocked, and at the same time, the spinal dorsal horn cells can inhibit the nociceptive stimulus-response, to play the pain treatment effect; and original “sick muscle theory.” Related studies show that FN treatment of postpartum pain and UI effect is significant.^[22]^

The pathophysiological causes of SUI mainly include maternal weight, uterine and fetal pelvic floor muscle trauma, collagen changes during pregnancy, hormonal changes during pregnancy, uterine expansion, and fetal weight.^[37]^ Therefore, for the rehabilitation of postpartum UI,^[13]^ conservative treatment of PFME or perineal rehabilitation is the first-line intervention for the treatment of postpartum UI. Compared with PFME, FN does not require women’s subjective efforts, mainly through the operator sweeping the affected muscle, to relieve pain and improve the function of the pelvic floor muscle. FN therapy has the advantages of simple operation, easy to form a standardized treatment plan, a relatively short course of treatment, and patients are not easy to fall off, which is greatly convenient for postpartum women, especially for patients who cannot exercise pelvic floor muscles. After floating acupuncture, the spatial configuration of loose connective tissue in a liquid crystal state was changed, which could conduct bioelectrical signals more efficiently. When the bioelectrical signal is applied to the diseased muscle or muscle group, it can relieve the spasm and stiffness of the rectus abdominis muscle and the femoris medial muscle group, increase the blood flow of the abdominal wall vein, the femoral vein, and the great saphenous vein, and the oxygen content of the rectus abdominis muscle and the femoris medial muscle group, to quickly relieve the pain.^[38]^ Through repeated reperfusion activities, the rectus abdominis muscle and femoris medial muscle group of ischemia repeatedly contract and relax, and internal blood circulation is accelerated, which improves muscle spasm and stiffness. When the strength of local pelvic floor muscles and urethral sphincter muscles is fully or partially restored, the sudden increase in bladder pressure can be completely or partially resisted, so as to achieve clinical cure or relief of SUI.^[39]^ Wang et al^27^ and Zhu et al^[24]^ conducted a study on the intervention of FN on postpartum pain and UI, which showed that FN therapy can effectively relieve the pain during delivery, help alleviate the symptoms of postpartum UI, and improve women’s postpartum quality of life. The study by Chen et al^[29]^ on the intervention of UI by FN combined with Kegel training also proved that FN therapy had a better effect in alleviating UI compared with Kegel training.

This systematic review also has some limitations: the quality of the study needs to be concerned; there may be some bias, thus reducing the accuracy and objectivity of the results; and the small sample size may reduce the effectiveness of the test. The intervention measures in this study were FN or FN combined therapy, and the combined results also could represent the overall efficacy of FN therapy, but interpreting the results requires caution. There are different diagnostic criteria and therapeutic indexes for postpartum pain, which also have a certain influence on the combined results. Most of the studies are short-term immediate effects and lack follow-up, so in the future, large sample, multicenter, high-quality RCTs are needed. It is necessary to further optimize the application of FN in postpartum pain and UI. The frequency of different FN sweeps, the difference in intervention time, the selection of acupoints/muscles for FN insertion, the diversity of the population, and the long-term efficacy are the main directions of future clinical FN research. In addition, no review protocol was registered in PROSPERO prior to conducting this study, which may introduce reporting bias. However, we adhered strictly to Preferred Reporting Items for Systematic reviews and Meta-Analyses guidelines to ensure transparency.

## 5. Conclusion

In summary, our meta-analysis and systematic review shows that FN therapy can alleviate postpartum pain and significantly reduce urinary leakage in postpartum patients, but FNs do not strengthen the pelvic floor muscles. This study, for the first time, provides evidence-based medical evidence for FN treatment of postpartum pain, improving UI and postpartum life quality. Based on what we have found, we encourage the use of FNs in clinical practice as one of the routine treatments for postpartum sequelae.

## Author contributions

**Conceptualization:** Dongmiao Han, Zicai Liu.

**Data curation:** Dongmiao Han, Min Tang, Zhitao Liu.

**Software:** Dongmiao Han, Zhitao Liu, Zicai Liu.

**Formal analysis:** Min Tang.

**Supervision:** Shuang Gong, Zicai Liu.

**Resources:** Wanyi Li.

**Methodology:** Zhitao Liu.

**Visualization:** Zicai Liu.

**Writing – original draft:** Dongmiao Han, Min Tang, Shuang Gong, Wanyi Li, Zhitao Liu, Zicai Liu.

**Writing – review & editing:** Dongmiao Han, Wanyi Li, Zicai Liu.
